# 3,5-Diethoxy-3′-Hydroxyresveratrol (DEHR) Ameliorates Liver Fibrosis via Caveolin-1 Activation in Hepatic Stellate Cells and in a Mouse Model of Bile Duct Ligation Injury

**DOI:** 10.3390/molecules23112833

**Published:** 2018-10-31

**Authors:** Phil Jun Lee, Hye-Jin Park, Namki Cho, Hong Pyo Kim

**Affiliations:** 1 College of Pharmacy, Ajou University, Suwon 16499, Korea; phil@ajou.ac.kr (P.J.L.); hyejin133@ajou.ac.kr (H.-J.P.); 2 Ilsong Institute of Life Science, Jung-gu, Gwan-yangdong 431-060, Korea; 3 College of Pharmacy and Research Institute of Drug Development, Chonnam National University, Gwangju 61186, Korea

**Keywords:** hepatic stellate cell (HSC), 3,5-diethoxy-3′-hydroxyresveratrol (DEHR), caveolin-1 (CAV1), heme oxygenase 1 (HO-1)

## Abstract

Hepatic stellate cells (HSCs) are involved in the pathogenesis of liver fibrosis. Resveratrol, 3,5,4′-trihydroxystilbene, is a dietary polyphenol found in natural food products. Here, we evaluated the anti-proliferative effects of a synthetic resveratrol derivative, 3,5-diethoxy-3′-hydroxyresveratrol (DEHR), on HSCs. Flow cytometry and Western blot analyses showed that DEHR induces apoptosis through the upregulation of cleaved caspase-3 and poly (ADP-ribose) polymerase expression and reduction in the level of an anti-apoptotic protein B-cell lymphoma 2 (Bcl2). As caveolin-1 (CAV1), a competitive inhibitor of heme oxygenase 1 (HO-1), is related to apoptotic proteins in hepatic cells, we focused on the role of CAV1 in DEHR-induced apoptosis in HSCs through Western blot analyses. Our results showed that the inhibitory effect of DEHR on cell viability was stronger in HO-1 siRNA-transfected cells but weakened in CAV1 siRNA-transfected cells. Collagen concentration was significantly reduced, whereas CAV1 expression increased after treatment of a bile duct ligation injury-induced liver fibrosis model with DEHR for four weeks. We confirmed that DEHR treatment significantly reduced fibrous hyperplasia around the central veins, using hematoxylin and eosin and Sirius red staining. DEHR ameliorates liver fibrosis in vitro and in vivo, possibly through a mechanism involving CAV1.

## 1. Introduction

Liver fibrosis is a severe disease caused by chronic liver injury in response to oxidative stress, viral infection, and biliary disorders [1]. The activation of hepatic stellate cells (HSCs) is a key event in the pathogenesis of liver fibrosis, leading to the accumulation of excess extracellular matrix (ECM) proteins, such as collagen [1]. Liver fibrosis has been perceived as an irreversible disease [2]. Recent studies have shown that death of activated HSCs may contribute to the termination of fibrogenesis during the resolution of liver fibrosis [2,3].

Caveolins are plasma membrane rafts present in most cells. Caveolin-1 (CAV1) protein is the principal component of the caveolin family. It has been linked with various cellular processes, including hepatic lipid homeostasis, vesicular transport, and signal transduction pathways [4,5,6]. CAV1 inhibits epidermal growth factor tyrosine kinase, extracellular signal-regulated kinase, threonine protein kinase, serine protein kinases, including Src family tyrosine kinase, protein kinase C alpha (PKCα), and H-Ras, via the CAV1 scaffolding domain [6,7,8,9]. Several studies attempted to develop phytochemicals with the potential of regulating CAV1 expression in various cell lines [10,11,12]. Recent reports evaluating the pathogenesis of liver fibrosis have suggested that CAV1 may play an important role in chronic liver disease [2,4,5].

Resveratrol (3,5,4′-trihydroxystilbene) is a dietary polyphenol in grapes, berries, nuts, and other various food products. The compound has been associated with the beneficial effects of red wine consumption [13]. Results of studies that explored the inhibitory effects of resveratrol on the proliferation of HSCs have implicated resveratrol as a promising candidate for the treatment of liver fibrosis [14,15,16,17]. Other studies have sought to improve the bioactivity of resveratrol [18,19,20]. For instance, trimethylated resveratrol is up to 100-fold more cytotoxic than normal resveratrol in vitro [18,20].

Heme oxygenase-1 (HO-1) is an antioxidant and cytoprotective protein that may be beneficial for the growth and survival of cells [21]. As HO-1 is associated with cell proliferation, drugs capable of regulating HO-1 expression are considered potential therapeutic candidates for the treatment of liver fibrosis [2,21]. A previous study reported that CAV1 is a competitive inhibitor of HO-1 and may function as an endogenous modulator of HO-1 [22]. CAV1 expression reportedly regulated the cytotoxic and pro-apoptotic activities of resveratrol in dose- and time-dependent manners in a hepatocellular carcinoma animal model [21].

In the present study, we investigated the cytotoxic effect of a synthetic resveratrol derivative, 3,5-diethoxy-3′-hydroxyresveratrol (DEHR), in HSCs in vitro. In addition, we evaluated the role of CAV1 and HO-1 on apoptotic cell death induced by DEHR. We further investigated whether DEHR exhibited anti-fibrotic activity in vivo in a bile duct ligation (BDL) mouse model of hepatic fibrosis. We determined CAV1 expression and collagen concentration in the BDL-induced liver fibrosis model after treatment with DEHR.

## 2. Results and Discussion

### 2.1. DEHR Induces Apoptosis in Activated HSCs

The activation of HSCs, which are a major source of ECM, is a key event in the pathogenesis of liver fibrosis (Bataller and Brenner, 2005). Several attempts have sought to discover agents from natural sources that contribute to the resolution of liver fibrosis through the inhibition of activated HSCs [1,2]. Evidence suggests that resveratrol, a naturally occurring chemical compound found in grapes, berries, nuts, and other food products, induces apoptosis of activated HSCs [14,15,16,17].

**Figure 1:**
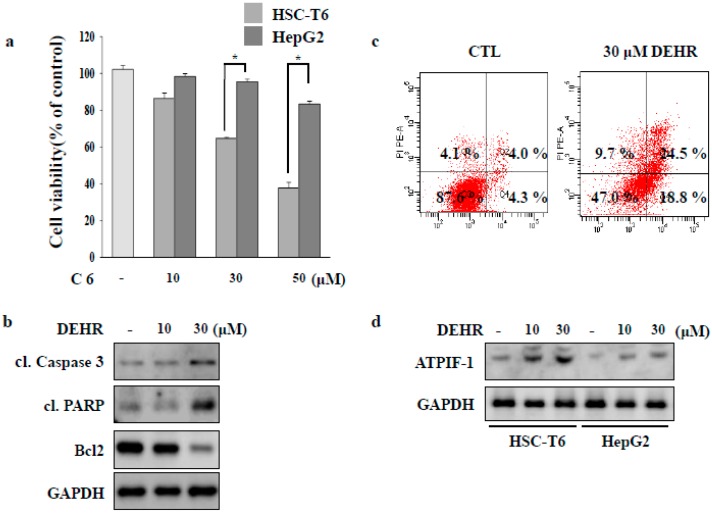
3,5-diethoxy-3′-hydroxyresveratrol (DEHR) inhibits the proliferation of activated hepatic stellate cells (HSCs). (**a**) HSC-T6 and HepG2 cells were treated with DEHR, and the MTT assay was performed to assess cell viability. (**b**) The expression levels of cleaved poly (ADP-ribose) polymerase (PARP), cleaved caspase-3, and Bcl2 were analyzed by Western blotting. Glyceraldehyde-3-phosphate dehydrogenase (GAPDH) served as a loading control. (**c**) Cells were incubated with 30 μM of DEHR for 24 h and subsequently stained with Annexin-V/PI. (**d**) The expression of ATPIF-1 was evaluated by Western blotting using GAPDH as a loading control. Data are representative of three independent experiments and expressed as mean ± SD (*n* = 3), * *p* < 0.05.

We investigated whether DEHR induces apoptosis in HSC-T6 cells via CAV1 and attenuates liver fibrosis in a BDL-induced mouse model. To evaluate the cytotoxic effect of synthetic resveratrol derivatives (Figure S1), HSCs were treated with 10 different compounds. Of these, compound 6 had notable inhibitory effect and good selectivity at 10, 30, and 50 µΜ (Figure 1A). The structural differences between DEHR and resveratrol include the presence of a diethoxy group at the meta-position of the A ring of resveratrol as well as the presence of 3′,4′-dihydroxy in the B ring (Figure S1). To investigate whether DEHR induces apoptosis in HSCs, we examined the apoptotic effect of DEHR using flow cytometry and Western blot analyses.

Cellular damage upon treatment with DEHR was accompanied by the upregulated expression of pro-apoptotic proteins cleaved caspase-3 and cleaved poly (ADP-ribose) polymerase (PARP), and reduction in the expression level of the anti-apoptotic protein Bcl2 (Figure 1B). Flow cytometry analysis of damaged cells probed with Annexin-V/PI demonstrated that the cells underwent apoptosis following incubation with DEHR (Figure 1C). The lower right quadrant (Annexin-V+/PI−) and the upper right quadrant (Annexin-V+/PI+) of the figure present the percentage of cells in early and late apoptosis, respectively. We further investigated the expression of ATPIF-1 that was increased following DEHR treatment (Figure 1D), possibly accounting for the impairment in the mitochondrial membrane potential by reducing ATP production [23]. Taken together, our findings show that the induction of apoptosis in HSC-T6 cells was prominent following treatment with DEHR.

### 2.2. Inhibition of Cytoprotective Proteins HO-1 and p62 Contributes to Apoptotic Cell Death by DEHR

**Figure 2:**
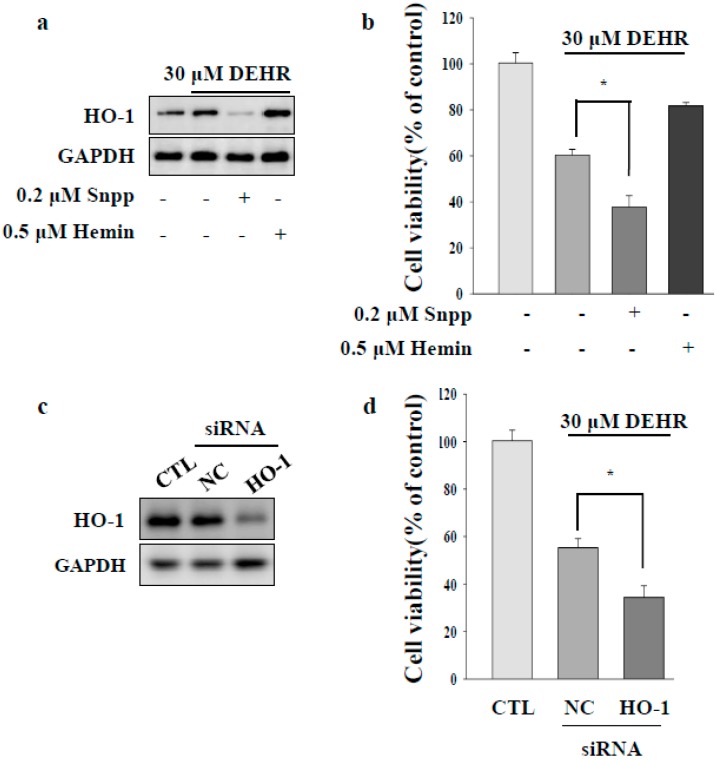
Inhibition of the cytoprotective protein HO-1 contributes to apoptotic cell death by DEHR. (**a**) Expression of HO-1 was analyzed by Western blotting. (**b**) To elucidate the HO-1 signaling pathway involved in DEHR-induced apoptosis of HSCs, a specific inhibitor of SnPP (tin protoporphyrin) and an inducer of hemin were used. HSCs were treated with DEHR, and the MTT assay was performed to assess cell viability. To confirm these findings, cells were transfected with siHO-1 and treated with DEHR. (**c**) Expression of HO-1 was analyzed by Western blotting. (**d**) The MTT assay was performed to assess cell viability. Data are representative of three independent experiments and expressed as mean ± SD (*n* = 3), * *p* < 0.05.

**Figure 3:**
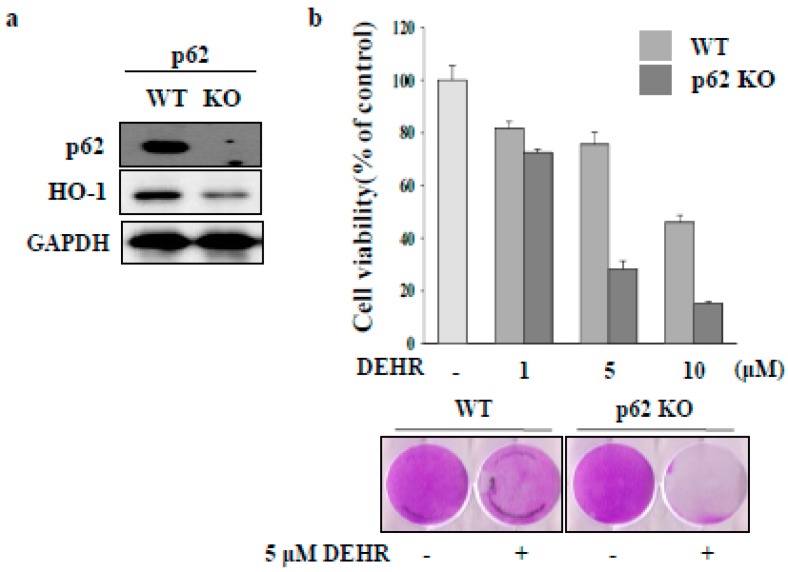
Inhibition of the cytoprotective protein p62 contributes to apoptotic cell death by DEHR. (**a**) HO-1 expression was detected in p62 wild-type cells. (**b**) The MTT assay was performed to assess cell viability in cells treated with DEHR. Data are representative of three independent experiments and expressed as mean ± SD, * *p* < 0.05.

HSC proliferation is dependent on the activity of HO [24]. Induction of HO-1 expression confers cytoprotection against noxious stimuli [25]. To investigate the involvement of the HO-1 signaling pathway in DEHR-induced apoptosis of HSCs, a specific inhibitor of SnPP (tin protoporphyrin) and an inducer of hemin were used (Figure 2A,B). Inhibition of HO-1 protein increased the cytotoxicity of DEHR, whereas induction of HO-1 expression resulted in the reduction in the cell death mediated by DEHR. To confirm these results, cells were transfected with siHO-1 and treated with DEHR (Figure 2C). An inhibitory effect of DEHR on cell viability was evident in the HO-1 siRNA-transfected cells (Figure 2D). As p62 has an important role in the separation of Nrf2 from Kelch-like ECH-associated protein 1 (Keap1) in an HO-1-dependent pathway [26], we investigated the expression of p62 (Figure 3). We confirmed that p62-ablated murine embryonic fibroblasts showed reduced expression of HO-1 (Figure 3A). Furthermore, compared with the wild-type group, the p62-knockout group showed increased cytotoxicity of DEHR (Figure 3B). These results show that the inhibition of the cytoprotective proteins HO-1 and p62 results in apoptotic cell death by DHER.

### 2.3. CAV1 Contributes to Apoptotic Cell Death by DEHR

**Figure 4:**
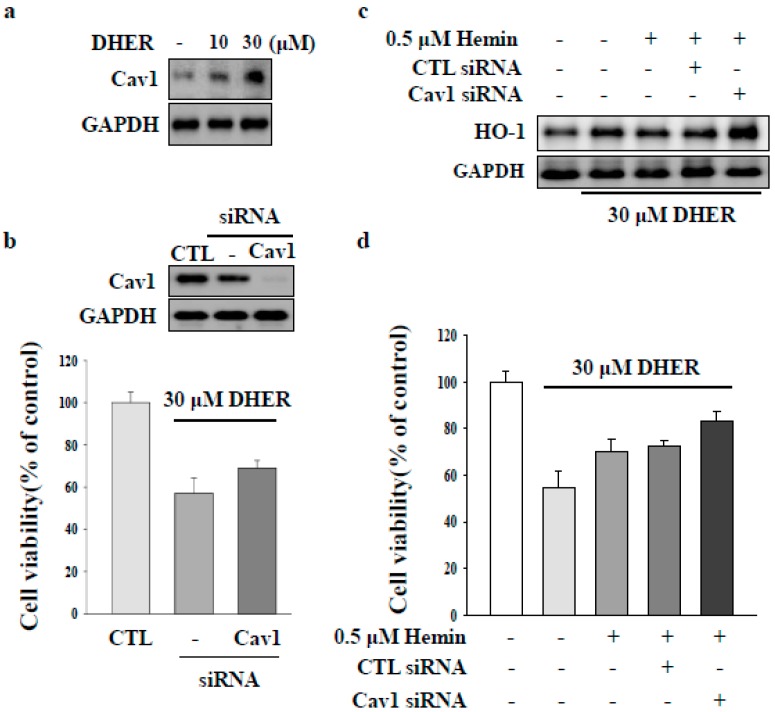
Caveolin-1 (CAV1) contributes to apoptotic cell death by DEHR. (**a**) The expression of CAV1 after treatment of cells with DEHR was analyzed by Western blotting. (**b**) Cells transfected with siCAV1 and treated with DEHR. (**c**) DEHR-induced apoptosis of HSCs decreased following treatment with the specific HO-1 inducer, hemin. The expression of HO-1 was analyzed by Western blotting. (**d**) MTT assay was performed to assess cell viability. Data are representative of three independent experiments and expressed as mean ± SD.

CAV1 protein is associated with the pathogenesis of liver fibrosis in chronic liver disease [2,4,5]. CAV1 is a competitive inhibitor of HO-1 [22]. In the present study, we focused on the role of CAV1 in DEHR-induced apoptosis of HSCs. DEHR treatment resulted in the upregulation of CAV1 expression in a dose-dependent manner (Figure 4A). In addition, transfection of cells with siCAV1 resulted in the inhibition of CAV1 and subsequently blocked DEHR-induced apoptosis of HSCs (Figure 4B). In line with the results shown in Figure 3, DEHR-induced apoptosis of HSCs decreased following treatment with the specific HO-1 inducer, hemin (Figure 4C). We also confirmed that cells transfected with siCAV1 showed a slight increase in the expression of HO-1, as evident from the last lane in Figure 4C. Our results suggest that CAV1 contributes to the apoptotic cell death effect of DEHR in activated HSCs and that CAV1 may serve as a competitive inhibitor of HO-1. Further studies are warranted to evaluate the precise role of CAV1 in the suppression of fibrogenesis.

**Figure 5:**
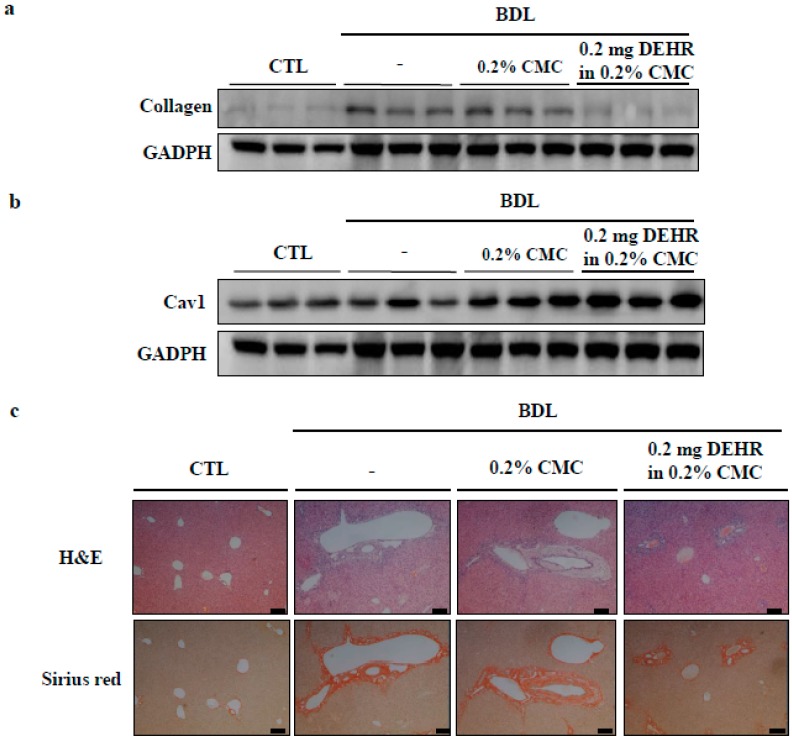
DEHR ameliorates BDL-induced liver fibrosis. DEHR was given per os at a dose of 0.2 mg/kg every day for 4 weeks (*n* = 6). Collagen content (**a**) and CAV1 expression (**b**) in liver sections were measured by Western blotting. (**c**) Liver damage was analyzed with hematoxylin and eosin and Sirius red staining.

To determine whether DEHR reduces the severity of hepatic fibrosis in an animal model, hepatic fibrosis was induced in a mouse BDL model of hepatic fibrosis for a period of four weeks. The content of hepatic collagen, a marker of fibrosis, is shown in Figure 5. At week four, the collagen concentration in BDL mice abruptly increased [27]. This finding was consistent with the enlarged spleen and bile in the gall bladder (Figure S2), suggesting the success of the surgery in establishing hepatic fibrosis. The increased collagen concentration in BDL mice was significantly decreased following treatment with DEHR at a dose of 0.2 mg/kg body weight (Figure 5A). Furthermore, the decrease in the expression of CAV1 in BDL mice was restored by DEHR treatment for four weeks (Figure 5B). Hematoxylin and eosin staining was performed to investigate the presence of fibrous hyperplasia (Figure 5C). DEHR treatment significantly reduced fibrous hyperplasia around the central veins. These results indicate that DEHR ameliorates liver fibrosis in vitro and in vivo by decreasing collagen concentration and activating CAV1 expression.

Reportedly, a dose of 27 µg/kg person is administered for resveratrol in prevention and treatment of common clinical conditions aging [28]. Based on this fact, same concentration of DEHR will be treated to clinical patient with hepatic fibrosis. However, because DEHR was resveratrol analogues, optimal concentration may be different for clinical trial. Also, a preferred concentration of drug is dependent on disease. Sequentially, optimal concentration of DHER will have to screen for oral administration.

## 3. Materials and Methods

### 3.1. Cell and Tissue Cultures

An immortalized rat HSC line was provided by Professor S.H. Sung (Seoul National University, Seoul, Korea). p62-knockout mouse embryonic fibroblasts (MEFs) were provided by Professor J.G Sin (Sungkyunkwan University, Suwon, Korea). HSCs and p62-knockout MEF were cultured according to KCLB directions in Dulbecco’s modified Eagle’s medium supplemented with 10% fetal bovine serum and 1% streptomycin (pen-strep) in an atmosphere of humidified 5% CO_2_ at 37 °C.

### 3.2. Cell Viability Assay

The cell viability of activated HSCs was determined by the 3-(4,5-dimethylthizaol-2-yl)-2,5-diphenyltetrazolium bromide (MTT) assay. In each experiment, cells were plated in 100 µL aliquots in growth medium in 96-well plates (10^5^ cells/well) and incubated for 24 h. DEHR was added to each well at concentrations of 10, 30, and 50 μΜ. MTT solution (5 mg/mL) was then added to each well and the formazan precipitate that developed during the incubation of cells for 2 h was dissolved in 100 µL of dimethyl sulfoxide. After incubation, the absorbance was measured using an automated microplate reader (Bio-Tek, Winnoski, VT, USA) at a wavelength of 562 nm. The relative cell survival (%) was calculated as a ratio between the absorbance of the treated and control (untreated) cells. The experiments were performed at least three times with each condition plated in triplicate.

### 3.3. Apoptosis Assay

Cells were incubated for various times following treatment with DEHR. Cells were detached with ethylenediaminetetraacetic acid (EDTA)-free trypsin and washed twice with cold phosphate-buffered saline (PBS). Cells were re-suspended in 400 µL of 1× loading buffer containing 5 µL Annexin-V and 5 µL of propidium iodide (PI; Becton-Dickinson, Santa Clara, CA, USA) for 15 min on ice in the dark. Analyses were performed using a FACSCalibur analyzer (Becton-Dickinson, San Diego, CA, USA).

### 3.4. Western Blotting and Antibodies

Cells were lysed with radioimmunoprecipitation (RIPA) assay buffer containing 1× PBS, 1% (*v*/*v*) Nonidet P-40 (NP-40), 0.5% (*w*/*v*) sodium deoxycholate, 0.1% (*w*/*v*) sodium dodecyl sulfate (SDS), 0.1 mg/mL phenylmethylsulfonyl fluoride, 30 μL/mL aprotinin, and 1 mM sodium orthovanadate. Cell lysates were centrifuged and the resulting supernatants were collected. Proteins were separated by 8%–15% SDS–polyacrylamide gel electrophoresis and transferred onto a polyvinylidene difluoride membrane. Each membrane was blocked in Tris-buffered saline containing 0.1% Tween-20 (TBST) and 5% non-fat dry milk for 1 h at room temperature, followed by an overnight incubation with a primary antibody in TBST containing 1% non-fat dry milk at 4 °C. Anti-cleaved caspase-3, anti-cleaved poly (ADP-ribose) polymerase (PARP), anti-B-cell lymphoma 2 (Bcl2), anti-ATPase inhibitory factor (ATPIF)-1, anti-HO-1, anti-p62, anti-CAV1, anti-collagen, and anti-glyceraldehyde phosphate dehydrogenase (GAPDH) were purchased from Santa Cruz Biotechnology (Santa Cruz, CA, USA). Membranes were washed with TBST and incubated with goat anti-rabbit or anti-mouse horseradish peroxidase-conjugated IgG secondary antibody for 2 h. Signal was measured using the chemiluminescence system (GE Healthcare, Piscataway, NJ, USA).

### 3.5. Mice

C57BL/6 mice (5 weeks old) purchased from DBL (Yumseng, Korea) were housed under a standard specific pathogen-free environment with a 12 h dark/light cycle and free access to water and food. All animal experiments were conducted in accordance with the Guidelines for the Care and Use of Laboratory Animals and were approved by the Animal Ethics Review Committees of Ajou University (Permission number: 2013–0006). Mice were divided into four groups: Sham group (*n* = 6), control group (*n* = 6), BDL group treated with 0.2% carboxymethylcellulose (CMC) (*n* = 6), and BDL group treated with DHER in 0.2% CMC (*n* = 6). Mice in the BDL group were orally administered with 0.2 mg/kg of DEHR suspended in 0.1% CMC every day for 4 weeks.

### 3.6. Bile Duct Ligation (BDL) Procedure

BDL surgeries were performed under anesthesia using an intraperitoneal injection of xylazine and ketamine at 10 and 100 mg/kg, respectively, which maintained the mice in a deeply anesthetized state for over 2 h while surgeries were performed. The surgical area was shaved and prepped by scrubbing the skin three times with alternating exposure to povidone-iodine and 70% ethanol. These surgeries involved a midline laparotomy (approximately 15 mm), followed by ligation of the bile duct with a silk thread at two positions to close the wounds. No adverse effect was observed. To test the effect of DEHR on BDL-induced hepatic fibrosis, oral administration (0.2 mg/kg) of the drug was performed for 4 weeks.

### 3.7. Histological Analysis

Sections (4-µm thick) from the right lobe of the liver were routinely processed for hematoxylin and eosin staining. For the detection of collagen fibers, these sections were similarly stained and were also stained with Sirius red [27].

### 3.8. Statistical Analyses

Statistical analyses were done using SPSS 22.0 software (SPSS Japan Inc., Tokyo, Japan). A two-tailed Student’s *t*-test was used for the analysis of continuous variables. We determined the differences among more than three groups using non-repeated measures analysis of variance (ANOVA) and the Scheffe test. Results are expressed as the mean ± standard deviation, and *p* < 0.05 was considered to indicate statistical significance.

## 4. Conclusions

DEHR, a synthetic resveratrol, inhibited activated HSCs through the upregulation of the expression of pro-apoptotic proteins, including cleaved caspase-3 and cleaved PARP, and downregulation of the expression of the anti-apoptotic protein Bcl2. We investigated whether HO-1 and p62 contribute to apoptotic cell death induced by DEHR in HSCs. The anti-fibrotic activity of DEHR seemed to be mediated via the induction of CAV1 signaling in vitro as well as in vivo. We confirmed that DEHR ameliorated BDL-induced liver fibrosis by decreasing the concentration of collagen. Although the precise roles played by DEHR in the suppression of fibrogenesis are yet unclear, our findings suggest that DEHR may be a candidate agent to resolve liver fibrosis likely through a mechanism that involves CAV1.

## Supplementary Materials

The supplementary materials are available online.
