# Supplementary figures and images for: 3,5-Diethoxy-3′-Hydroxyresveratrol (DEHR) Ameliorates Liver Fibrosis via Caveolin-1 Activation in Hepatic Stellate Cells and in a Mouse Model of Bile Duct Ligation Injury
Source: Molecules. 2018 Oct 31;23(11):2833. doi: 10.3390/molecules23112833 (PMC6278252; doi:10.3390/molecules23112833)

A

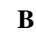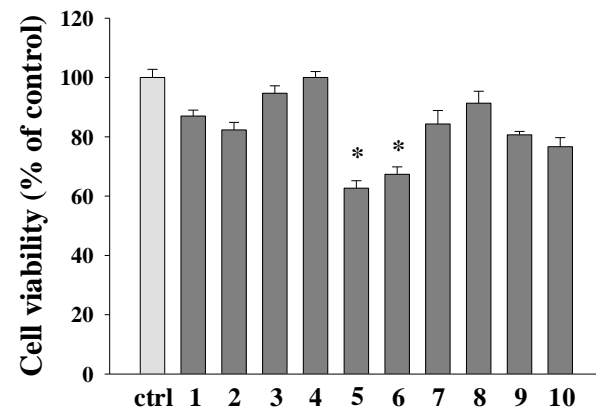

**Figs 2. BDL-induced liver fibrosis.**

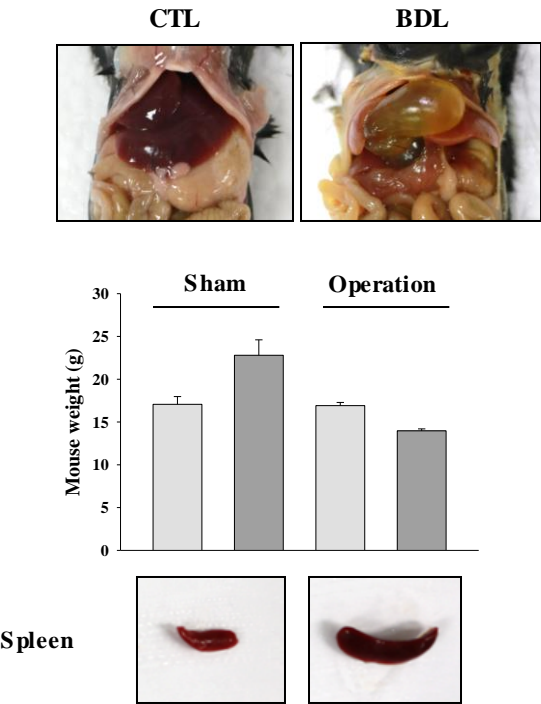

Supplement: Supplementary file 1 [file molecules-23-02833-s001.zip › Supplymental Figure.pdf]
